# Brazilian Dialysis Survey 2020

**DOI:** 10.1590/2175-8239-JBN-2021-0198

**Published:** 2022-02-23

**Authors:** Fabiana B Nerbass, Helbert do Nascimento Lima, Fernando Saldanha Thomé, Osvaldo Merege Vieira, Jocemir Ronaldo Lugon, Ricardo Sesso

**Affiliations:** 1 Fundação Pró-Rim, Joinville, SC, Brasil.; 2 Universidade da Região de Joinville, Joinville, SC, Brasil.; 3 Universidade Federal do Rio Grande do Sul, Porto Alegre, RS, Brasil.; 4 Universidade de São Paulo, Ribeirão Preto, SP, Brasil.; 5 Universidade Federal Fluminense, Niterói, RJ, Brasil.; 6 Universidade Federal de São Paulo, São Paulo, SP, Brasil.

**Keywords:** Renal Dialysis, Peritoneal dialysis, Peritoneal Dialysis, Epidemiology, COVID-19, Diálise Renal, Diálise Peritoneal, Epidemiologia, COVID-19

## Abstract

**Introduction:**

National data on chronic dialysis treatment are essential to support the development of health policies aimed at improving the treatment for thousands of people.

**Objective:**

To report epidemiological data from the 2020 Brazilian Dialysis Survey, sponsored by the Brazilian Society of Nephrology.

**Methods:**

A survey was carried out in Brazilian chronic dialysis centers using an online questionnaire for the year, covering clinical and epidemiological aspects of patients in a chronic dialysis program, data on dialysis therapy, characteristics of dialysis units and the impact of the COVID-19 pandemic.

**Results:**

235 (28%) of the centers responded to the questionnaire. In July 2020, the estimated total number of patients on dialysis was 144,779. The estimated prevalence and incidence rates of patients per million population (pmp) were 684 and 209, respectively. Of the prevalent patients, 92.6% were on hemodialysis (HD) and 7.4% were on peritoneal dialysis (PD); 23% were on the transplant waiting list. A central venous catheter was used by a quarter of patients on HD. The incidence rate of confirmed COVID-19 between February and July 2020 was 684/10,000 dialysis patients, and the lethality rate was 25.7%. The estimated overall mortality and COVID-19 crude annual mortality rates were 24.5 and 4.2%, respectively.

**Conclusion:**

The absolute number of patients on chronic dialysis and prevalence rate continued to increase. The low use of PD as dialysis therapy was maintained and the use of long-term catheters for HD increased. The COVID-19 pandemic contributed to the increase in the overall mortality rate.

## Resumo

**Introdução:**

Dados nacionais sobre tratamento dialítico crônico são essenciais para apoiar o desenvolvimento de políticas de saúde destinadas a melhorar o tratamento de milhares de pessoas.

**Objetivo:**

Relatar dados epidemiológicos do Censo Brasileiro de Diálise 2020, patrocinado pela Sociedade Brasileira de Nefrologia.

**Métodos:**

Realizou-se pesquisa em centros brasileiros de diálise crônica utilizando questionário online relativo ao ano, abrangendo aspectos clínicos e epidemiológicos de pacientes em programa de diálise crônica, dados sobre terapia dialítica, características das unidades de diálise e impacto da pandemia COVID-19.

**Resultados:**

235 (28%) centros responderam ao questionário. Em julho de 2020, o número total estimado de pacientes em diálise foi 144.779. As taxas estimadas de prevalência e incidência de pacientes por milhão da população (pmp) foram 684 e 209, respectivamente. Dos pacientes prevalentes, 92,6% estavam em hemodiálise (HD) e 7,4% em diálise peritoneal (DP); 23% estavam na lista de espera para transplante. Cateter venoso central foi utilizado por um quarto dos pacientes em HD. A taxa de incidência de COVID-19 confirmada entre fevereiro e julho de 2020 foi 684/10.000 pacientes em diálise, a taxa de letalidade foi 25,7%. As taxas estimadas de mortalidade geral e mortalidade bruta anual por COVID-19 foram 24,5 e 4,2%, respectivamente.

**Conclusão:**

O número absoluto de pacientes em diálise crônica e a taxa de prevalência continuaram a aumentar. O baixo uso de DP como terapia de diálise foi mantido e o uso de cateteres de longa permanência para HD aumentou. A pandemia COVID-19 contribuiu para o aumento da taxa de mortalidade geral.

## Introduction

With the aim of obtaining and analyzing data on clinical and epidemiological aspects of patients undergoing chronic dialysis, in addition to information on dialysis therapy, the Brazilian Society of Nephrology (BSN) annually sponsors the Brazilian Dialysis Survey^1,2^. Since its initial implementation in 1999, the survey has been conducted nationwide and, in the last decade, it has been completed electronically by dialysis centers. The continuous survey is justified because it provides relevant information for the development of health policies and strategies aimed at improving care for individuals undergoing dialysis. In this study we are reporting data from the 2020 Brazilian Dialysis Survey, including information about the impact of the COVID-19 pandemic on dialysis clinic patients and staff.

## Methods

### Data collection

Dialysis clinics filled out an online questionnaire available on the BSN website. It contained questions about sociodemographic, clinical, and therapeutic variables of patients on chronic dialysis (Supplement). The questionnaire was available from August 2020 to January 2021. Participation in the survey was voluntary, and all dialysis centers registered at BSN were invited to participate by email and by BSN media. After the initial invitation, reminders were emailed monthly to centers that had not filled the questionnaire. During the survey period, the chairs of the regional sections of the BSN were asked to contact the dialysis centers in their states and reiterate the importance of their participation.

### Data analysis

Data for each center were grouped rather than analyzed individually. For the 2020 survey, 235 of 834 active centers responded to the questionnaire, a response rate of 28%.

For national estimates of total number of patients and prevalence rate, the sample was expanded. We assumed that the units that did not respond to the questionnaire had the same number of patients as participants (mean of 173.6 patients per unit). Because this extrapolation can be imprecise, we used a variation of ± 5% in the obtained mean (164.9 to 182.3 patients per unit) for the prevalence calculations. Likewise, the mean number of new patients per unit was applied to units that did not report incidence rates. All other sociodemographic data and patient characteristics refer to the sample studied. Annual mortality and annual incidence of patients on dialysis were estimated from events in July 2020. For calculating prevalence and incidence rates, national and regional population data were obtained from the Brazilian Institute of Geography and Statistics (IBGE) estimates for July 2020. According to IBGE, the Brazilian population at that date was 211.75 million inhabitants^3^. Most data were presented descriptively, and results were compared with data from previous years. Information on COVID-19, such as incidence, hospitalizations, and lethality, was considered for the period from February 26^th^ (date of the first case reported in the country) to July 31^st^, 2020. The diagnosis of COVID-19 required confirmation by real-time polymerase chain reaction (RT-PCR) of nasal/oropharyngeal specimens or serologic testing.

### Calculations of estimates

The main calculations and estimates are shown in Table 1.

**Table 1:** Calculations of incidence, prevalence and mortality estimates

Estimates	Formula
Estimated total number (N) of patients on 1^st^ of July	N of patients in the sample/ proportion of participating centers
Estimated annual prevalence rate of dialysis patients (pmp)	Estimated total N of patients on 1^st^ of July / Brazilian population on 1^st^ of July^3^
Estimated total N of patients starting treatment	N of individuals starting treatment in July x 12 / proportion of active participating centers
Estimated annual incidence rate of dialysis patients (pmp)	Estimated total N of patients starting treatment / Brazilian population on 1^st^ of July^3^
Estimated total annual number of deaths	N of deaths reported in July x 12 / proportion of active participating centers
Estimated crude annual mortality rate (%)	Estimated total N of deaths in 2020 x 100/ Estimated N of dialysis patients on 1st of July
Estimated COVID-19 crude annual mortality rate (%)	Number of deaths due to Covid-19 from March to July 2020 x (12/5) x 100 / Estimated N of dialysis patients on 1^st^ of July

pmp: per million population.

## Results

### Estimated incidence, prevalence, and mortality rates

In July 2020, there were 834 active chronic dialysis centers registered at BSN, a number 3.6% higher than in 2019. However, the percentage of participating centers decreased compared to the previous year (from 39 to 28%), with a slight difference in percentage of participation among regions (North and Midwest: 25%; South: 28%; Northeast and Southeast: 29%). The lower adherence resulted in a 25% decrease in patients whose data were used for the annual report (54,488 to 40,795).

**Figure 1:**
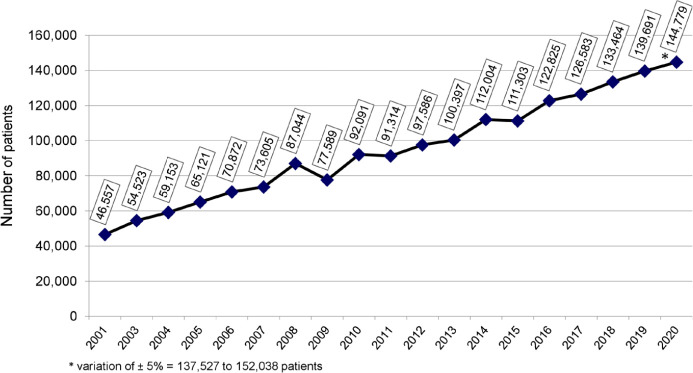
Estimated number of patients on chronic dialysis per year.

**Figure 2:**
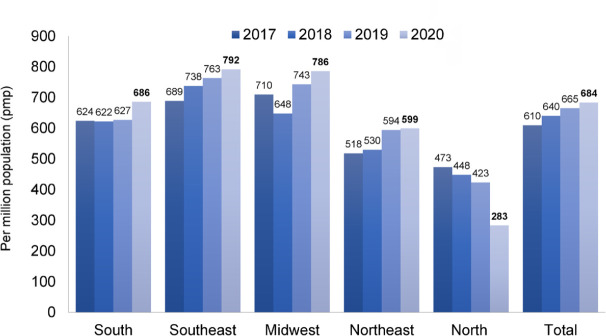
Estimated prevalence of patients on dialysis by geographic region in Brazil, per million population.

**Figure 3:**
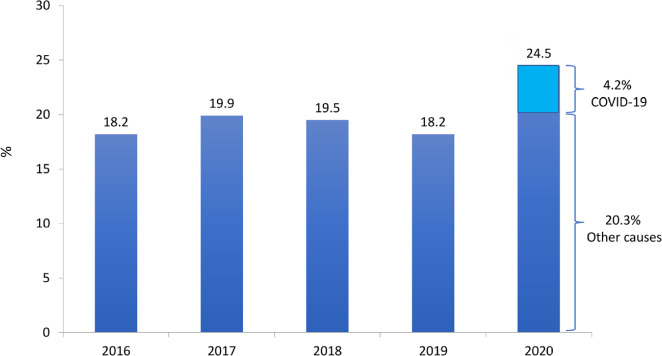
Estimated annual crude mortality rate of dialysis patients.

The estimated total number of patients in July 2020 was 144,779 (a variation of ± 5% = 137,527 to 152,038), 3.6% higher than July 2019. The trend observed in recent years toward an increase in number of dialysis patients continued in 2020 (Figure 1). The prevalence rate of dialysis patients also continued to increase, from 665 in 2019 to 684 per million population (pmp) in 2020, consistent with the trend observed in recent years. When stratified by region, a significant decrease in prevalence rate was observed only in the North region (Figure 2); the numbers increased slightly in the other regions. The estimated number of new dialysis patients in 2020 was 44,264, and the overall incidence rate was 209 pmp, lower than in 2019 when it reached 218 pmp. The incidence rate ranged from 75 pmp in the North region to 227 pmp in the South. The estimated number of deaths for the entire year was 35,413. The annual crude mortality rate has been between 18 and 20% since 2016, and is projected to increase to 24.5% in 2020 (Figure 3).

### Demographic and clinical characteristics

**Figure 4:**
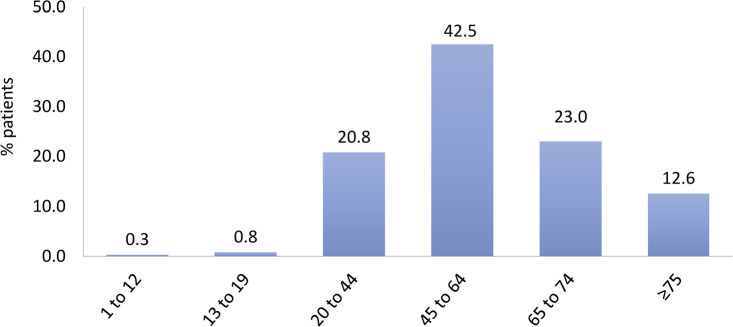
Distribution of patients according to age group.

**Figure 5:**
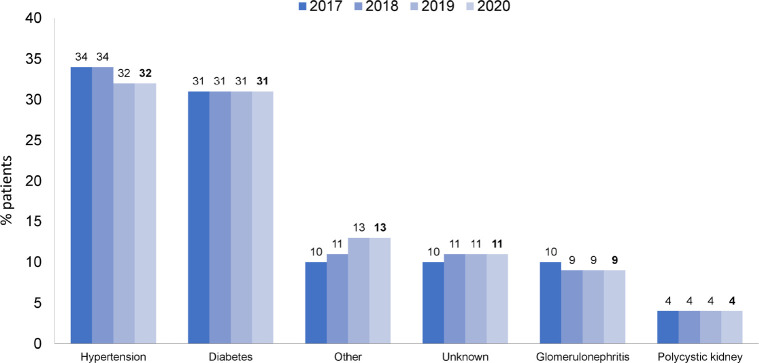
Distribution of dialysis patients according to chronic kidney disease etiology.

**Figure 6:**
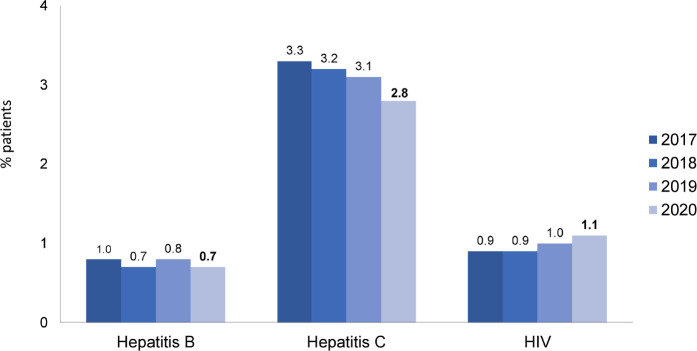
Prevalence of patients with positive serology for hepatitis B and C and HIV viruses.

**Figure 7:**
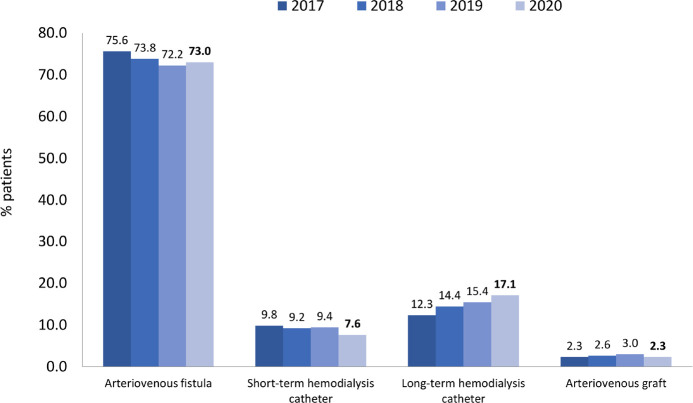
Distribution of vascular accesses used for hemodialysis.

The most prevalent age group was between 45 and 64 years, representing 42.5% (Figure 4). The distribution by sex, 58% men and 42% women, remained stable, as did the percentage of main underlying diseases. Systemic arterial hypertension and diabetes mellitus accounted each for almost one third of all cases (Figure 5). The percentage of patients with hepatitis C continued to decrease, while the percentage of patients with hepatitis B and HIV remained stable (Figure 6). Regarding vascular access, 25% of hemodialysis (HD) patients used a central venous catheter. A decrease in the use of short-term catheters and prostheses was observed, while the use of long-term catheters increased (11%) (Figure 7). The estimated number of dialysis patients on the kidney transplant waiting list in 2020 was 33,239 (23%), similar to the previous year.

### Characteristics of dialysis treatment

The distribution of patients by dialysis modality and payment source is shown in Table 2. Hemodialysis (HD) continued to be the treatment for most patients (92.6%) and 7.4% were treated with peritoneal dialysis (PD). Treatment was financed by the public health system for 81.6% of patients and by private health insurance for 18.4% of patients in the participating units.

**Table 2:** Distribution of patients by modality of dialysis and paying source

Modality	Public health		Private health		Total	
	N	%	N	%	N	%
HD ≤ 4 sessions/week	30,820	92,5	6,208	83,0	37,028	90,8
HD > 4 sessions/week	161	0,5	599	8,0	760	1,9
Home HD	0	0,0	7	0,1	7	0,0
CAPD	531	1,6	77	1,0	608	1,5
APD	1,786	5,4	587	7,8	2,373	5,8
IPD	19	0,1	0	0,0	19	0,0
Total	33,317	100	7,478	100	40,795	100

HD: hemodialysis; CAPD: continuous ambulatory peritoneal dialysis; APD: automated peritoneal dialysis; IPD: intermittent peritoneal dialysis.

### Characteristics of participating centers

Of the 235 participating dialysis units, 71% were privately owned, 18% were philanthropic, and 10% were public. Among private clinics, international corporations managed 14.4% of participating units. Most centers described themselves as satellite (54%), and 46% were in-patient units. Fifty-six percent of participating units reported having PD as a treatment option. The national average number of patients per nephrologist was 27 and ranged from 21 in the North to 30 in the South.

### COVID-19

**Figure 8:**
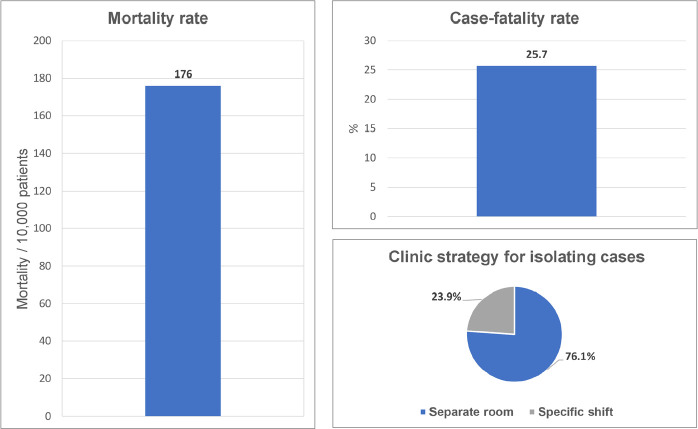
COVID-19 mortality rate, case-fatality rate and strategy adopted to isolate cases.

In the 234 dialysis centers between March and July, there were 2791 reported cases of COVID-19. The incidence rate of confirmed COVID-19 between February 26^th^ and July 31^st^, 2020 was 684/10,000 dialysis patients. Confirmation was performed by real time polymerase chain reaction (RT-PCR) in 68.4%, by serologic testing in 26.3%, and by both methods in 6%. Of the total number of infected patients, 95.7% were on HD and 4.3% on PD. Nearly 52% of confirmed cases were hospitalized and, of these, 57.6% required treatment in intensive care units. Seven hundred and eighteen COVID-19 patients died. Case-fatality rate was 25.7% and mortality rate reached 176/10,000 patients. The estimated crude annual mortality rate attributed to COVID-19 was 4.2%. As a strategy to isolate suspected or confirmed cases of COVID-19, 76.1% of participating centers reported treatment in a separate room or space. The remainder (23.9%) chose transfer to a specific shift (Figure 8). The total percentage of infection among health professionals working in clinics was 21.9%. The percentages in physicians, nurses, and nursing technicians were 25.1, 24.0, and 20.8%, respectively. Death by COVID-19 was only reported in nursing technicians (0.1%).

## Discussion

In the last two decades, the Brazilian dialysis survey has highlighted the panorama of dialysis treatment, providing data and analyses that contribute to public policies and strategies to improve this therapy in our country. In general, the trends observed in recent years were maintained in 2020. As a novelty, we reported information on the impact of the COVID-19 pandemic on dialysis centers.

There was a decrease in the percentage of dialysis units participating in the 2020 survey compared to the previous year, from 39 to 28%. Although we did not investigate the causes, we believe that the difficulties of the COVID-19 pandemic faced by dialysis centers may have contributed to this lower voluntary participation. Despite that, participation was around 30% and was similar across the five macro-regions, increasing the chance of national representativeness of the sample.

The upward trend in total number of patients by 3.6% and in prevalence by 2.9% followed the pattern observed in recent years. This may be explained by the increase in population longevity^3^ and by improvements in the quality of care and facilitation of dialysis access for patients with chronic kidney disease, although the latter should be confirmed by further investigations. Compared with the Latin American Society of Nephrology (SLAHN) 2018 registry^4^, our prevalence of people on dialysis (684 pmp) was slightly higher than the average of the included countries (617 pmp). Our numbers were substantially lower than those of the United States in 2017 (1538 pmp)^5^ and higher than those of the European registry in 2018 (556 pmp)^6^.

The 2020 incidence rate (209 pmp) was slightly lower than the national estimate in 2019 (218 pmp), higher than that of Latin America (159 pmp)^4^ and Europe (122 pmp) in 2018^6^ and lower than that of the United States (370 pmp) in 2017^5^.

We found a significant increase in crude mortality rate, which had been close to 20% in the last years, but reached 24.5% in 2020. The estimated annual number of deaths among dialysis patients jumped from 25,400 to 35,400. In addition to the increase in age and a greater burden of comorbidities in patients in recent years, the mortality associated with COVID-19 in dialysis^7^ may partially account for the finding. According to our estimate, the annual crude mortality rate attributed to COVID-19 was 4.2%, corresponding to 17.3% of the overall annual crude mortality rate (estimated at 25.7%). It is important to highlight that July and August were the most critical months of 2020 for the COVID-19 pandemic in hemodialysis^8^. Therefore, it is conceivable that the extrapolation of July mortality to 12 months led to an overestimation of the annual crude mortality rate for 2020. Regarding the distribution by age group, the upward trend in the prevalence of patients over 45 years of age continues, with those over 75 years of age accounting for 11.8% of the total, slightly more than half of the prevalence observed in developed countries^5,6,9^. The prevalence of male dialysis patients has remained constant at 58%.

A trend toward the stability of baseline diagnosis was observed, with hypertension and DM accounting for 32 and 31%, respectively. In the US in 2017, hypertension accounted for 30% and DM for 45% of baseline diagnoses of the dialysis population^5^.

The decrease in the percentage of patients with hepatitis C continued in 2020, falling below 3% for the first time. This result was a consequence of the implementation of preventive measures such as the reduction of blood transfusions and the prohibition of the reuse of dialyzers and lines for patients with positive serology, as well as the improved access to direct-acting antiviral agents, which allowed high cure rates^10^.

As in the previous year, it was found that approximately a quarter of HD patients (24.7%) used a central venous catheter as vascular access (7.6% short-term and 17.1% long-term). In the United States, the prevalence of central venous catheters has remained at one-fifth of all patients on HD therapy.^5^ There was an increase in the use of long-term catheters, which was 62% in 2019 and 69% in 2020.

Although more than half of the participating centers offer PD as treatment option (56%), only 7.4% of dialysis patients were treated by this modality of renal replacement therapy. The main reason seems to be the model proposed by our public health system, which is not economically viable for most clinics^11^. In some countries, strategies that have led to increased use of PD included the implementation of policies and incentives that favor this modality, allow the production and supply of materials at a low cost, and appropriate training for nephrology teams aimed increasing the use of therapy and continuously reducing the failure rates^12^.

We first reported that multinational companies operate about 15% of private dialysis centers. However, we emphasize that this share seems to be increasing in recent years and that the accuracy of our estimate may be limited due to sampling and the low response rate of these centers.

COVID-19 information limited to the period February 26-July 31, 2020, yielded an incidence rate of 684/10,000 patients. We emphasize that this rate was probably underestimated because it assumes a positive diagnostic test, which excludes many asymptomatic, untested cases. The lack of testing in the population, especially in the initial period of the pandemic, also influenced the result obtained. The high need for hospitalization for positive cases in intensive care units and the high lethality confirm the national and global results already reported^7,13-16^. Nevertheless, we believe that we found a high lethality rate because we included more severe patients in the sample and possibly because of the lack of experience in case management and the non-standardized treatment of patients in the first months of the pandemic.

We highlight as limitations the electronic data collection through voluntary completion, the aggregation of patient data by dialysis center, and the lack of response validation. Because only about 30% of active dialysis centers participated in the study, the methodology used in the national estimates of prevalence and incidence rates has limited accuracy, and caution should be used in interpreting the data. Our death rates estimates should also be interpreted with caution. The 2020 peak of COVID-19 cases in hemodialysis in July and August may have impacted our estimate of the overall annual crude mortality rate for 2020 because July figures were extrapolated to the year.

In conclusion, the 2020 survey confirmed a continuous increase in prevalence rate over the years and showed a slight decrease in incidence of dialysis patients in our country. The low prevalence of PD as dialysis therapy continues, although more than half of the centers offer this dialysis modality. The trend towards a progressive increase in the use of long-term central venous catheters was maintained. Also, the high lethality of COVID-19 on dialysis had an unfavorable impact on crude mortality rate in this population, and the influence of the COVID-19 pandemic in this population warrants continuous monitoring. Regular national assessment of patients on chronic dialysis is paramount for monitoring the quantitative and qualitative aspects of chronic kidney disease care in the country.

## Supplementary Material

The following online material is available for this article:

Supplementary Material - Formulary Census 2020 - Information needed to complete the online Census 2020.
