# Supplementary material for: Brazilian Dialysis Survey 2020
Source: J Bras Nefrol. 2022 Feb 23;44(3):349–57. doi: 10.1590/2175-8239-JBN-2021-0198 (PMC9518621; doi:10.1590/2175-8239-JBN-2021-0198)
Supplement: Supplementary Material - [file 2175-8239-jbn-2021-0198-suppl1.pdf]

Braz. J. Nephrol. (J. Bras. Nefrol.)
https://doi.org/10.1590/2175-8239-JBN-2021-0198

Supplementary Material to the "Brazilian Dialysis Survey 2020"

ATTENTION: You must fill out the form on the website, forms sent by e-mail or fax will not be considered.

INFORMATION NEEDED TO COMPLETE THE ONLINE

CENSUS 2020

2020 Census - Registration

STEP 01 - Registration

Registration information
Company Name:
CNPJ#:
Hospital:
Address:
City:
STATE:
ZIP:
Telephone:
Extension:
Fax:
E-mail:
Physician in Charge - Dr.:

Person responsible for filling this form up:
Capacity: ( ) Manager ( ) Social worker ( ) Nurse ( ) Physician ( ) Secretary

Number of Nephrologists working in the Dialysis Center:

Unit Profile Type: ( ) Non-profitable ( ) Private* ( ) Public

University? ( ) Yes ( ) No

If private:
Clinic owners:
( ) Group made up of nephrologists
( ) Group made up of nephrologists and non-nephrologist physicians
( ) Group made up of non-nephrologist physicians
( ) Group made up of physicians and non-medical personnel
( ) Hospital where the clinic is located
( ) International corporation

Localization: ( ) in-hospital ( ) remote

Partnerships
( ) Public Healthcare System (SUS) ( ) Other Healthcare Insurances ( ) Both

Dialysis

Is there a chronic dialysis program (HD or PD)?
(   ) yes   (   ) no

If yes:
Working?
(   ) yes   (   ) no

IF YOUR PROGRAM IS WORKING, PLEASE ANSWER THE FOLLOWING QUESTIONS; IF NOT, YOU HAVE FINISHED YOUR FORM.

STEP 02 – Demographic data

Registration information

Total number of patients under regular dialysis on July 01, 2020:

Number of patients under the chronic dialysis program on July 01, 2020:

	SUS	Non-SUS	Total
Conventional HD at the dialysis center			
Daily HD (more than 4 times a week) in the dialysis center			
Home HD, any regimen			
CAPD			
APD			
IPD			
Total			

***SUS: Public Healthcare System**

The following questions have to do with patients in the chronic dialysis program on July 01, 2020

Patient profiles:
Age range
0 to 12 years:
13 to 19 years:
20 to 44 years:
45 to 64 years:
65 to 74 years:
75 years or older:

Sex
Male:
Female:

Chronic kidney disease etiology: (Fill out Only 1 diagnostic per patient)

Diabetes:
Hypertension:
Glomerulonephritis:
Polycystic kidneys:
Others:
Undetermined:

Number of patients currently under dialysis who have received a kidney transplant and lost it:
Number of patients on HD using short-term central venous catheter as a hemodialysis access on Jul/01/2020.
Number of patients on HD using long central venous catheter as hemodialysis access on Jul/01/2020
Number of patients on HD using arteriovenous graft (prothesis) as hemodialysis access on Jul/01/2020:

Comorbidities:

Number of patients with:
B hepatitis:
C hepatitis:
HIV:

KIDNEY TRANSPLANT
Number of patients enrolled for transplant on Ju/01/2020:

STEP 03
New patients:
(Who started dialysis on July 2020 – DOES NOT INCUDE patients who came from another regular dialysis program; Only those who truly started a chronic dialysis program and were not on dialysis before).
Total:
Number of new patients diagnosed with diabetes:

Deaths:
Deaths in july 2020: (If none, enter 0)

STEP 04: Census about COVID-19 on dialysis
On this step we ask you to fill out information about the COVID-19 pandemic impacts on patients and employees of dialysis clinics. The questions below pertain to the period of <u>02/26/2020 to 07/31/2020</u>.

<u>As for the patients with Covid-19</u>
Number of patients with diagnosis confirmed by exam:_______;
Of the confirmed cases, how many were on hemodialysis? _____; How about on peritoneal dialysis? __________
Number of patients admitted to a hospital:
Among the in-patients, how many required intensive care?
Strategy used in the clinic concerning those patients with suspicion/diagnosis of COVID:
(   ) Dialysis under isolation: separated room/box
(   ) Dialysis on a specific shift
Confirmed number of deaths by Covid-19:
<u>Concerning healthcare professionals with Covid-19</u>
Number in the clinic: Physicians: _____ Nurses: _____ Nurse assistants: _____
Number with confirmed diagnosis: Physicians: _____ Nurses: _____ Nurse assistants: _____
Number of deaths: Physicians: _____ Nurses: _____ Nurse technicians: _____
